# (4,4′-Di-*tert*-butyl-2,2′-bipyridine-κ^2^ *N*,*N*′)bis­(nitrato-κ^2^ *O*,*O*′)copper(II)

**DOI:** 10.1107/S1600536809001457

**Published:** 2009-01-17

**Authors:** Xin Xiao, Zai-Ying Rao, Yun-Qiang Zhang, Sai-Feng Xue, Zhu Tao

**Affiliations:** a Key Laboratory of Macrocyclic and Supramolecular Chemistry of Guizhou Province, Guizhou University, Guiyang 550025, People’s Republic of China; b Institute of Applied Chemistry, Guizhou University, Guiyang 550025, People’s Republic of China

## Abstract

In the crystal of the title compound, [Cu(NO_3_)_2_(C_18_H_24_N_2_)], the Cu^II^ ion is coordinated by two N atoms of the bipyridine ligand and four O atoms from the two nitrate anions in a distorted octahedral fashion. The dihedral angle between the planes of the two pyridine rings is 11.52 (10)°. In the crystal structure, weak C—H⋯O inter­actions may help to establish the packing.

## Related literature

For general background, see: Noro *et al.* (2000 ▶); Yaghi *et al.* (1998 ▶); Huertas *et al.* (2001 ▶); Qin *et al.* (2002 ▶).
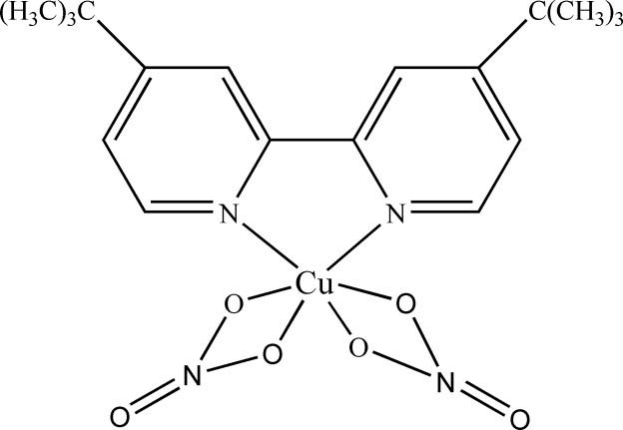


## Experimental

### 

#### Crystal data

[Cu(NO_3_)_2_(C_18_H_24_N_2_)]
*M* *_r_* = 455.96
Orthorhombic, 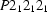

*a* = 9.8265 (16) Å
*b* = 13.247 (2) Å
*c* = 16.138 (3) Å
*V* = 2100.7 (6) Å^3^
*Z* = 4
Mo *K*α radiation
μ = 1.08 mm^−1^
*T* = 173 (2) K
0.27 × 0.25 × 0.17 mm

#### Data collection

Bruker SMART CCD area-detector diffractometer
Absorption correction: multi-scan (*SADABS*; Bruker, 2005 ▶) *T*_min_ = 0.759, *T*_max_ = 0.837
11065 measured reflections
3642 independent reflections
3450 reflections with *I* > 2σ(*I*)
*R*_int_ = 0.022

#### Refinement

*R*[*F* ^2^ > 2σ(*F* ^2^)] = 0.030
*wR*(*F* ^2^) = 0.074
*S* = 1.05
3642 reflections
258 parameters
1 restraint
H-atom parameters constrained
Δρ_max_ = 0.64 e Å^−3^
Δρ_min_ = −0.81 e Å^−3^
Absolute structure: Flack (1983 ▶), 1522 Friedel pairs
Flack parameter: 0.012 (13)

### 

Data collection: *SMART* (Bruker, 2002 ▶); cell refinement: *SAINT* (Bruker, 2002 ▶); data reduction: *SAINT*; program(s) used to solve structure: *SHELXS97* (Sheldrick, 2008 ▶); program(s) used to refine structure: *SHELXL97* (Sheldrick, 2008 ▶); molecular graphics: *ORTEP-3 for Windows* (Farrugia, 1997 ▶); software used to prepare material for publication: *WinGX* (Farrugia, 1999 ▶).

## Supplementary Material

Crystal structure: contains datablocks global, I. DOI: 10.1107/S1600536809001457/at2708sup1.cif

Structure factors: contains datablocks I. DOI: 10.1107/S1600536809001457/at2708Isup2.hkl

Additional supplementary materials:  crystallographic information; 3D view; checkCIF report

## Figures and Tables

**Table 1:** Hydrogen-bond geometry (Å, °)

*D*—H⋯*A*	*D*—H	H⋯*A*	*D*⋯*A*	*D*—H⋯*A*
C1—H1⋯O4^i^	0.93	2.46	3.124 (3)	129

Symmetry code: (i) 
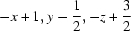
.

**Fig. 1.**
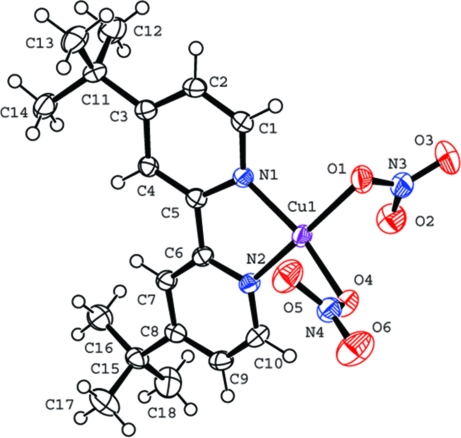
The molecular structure of (I) showing the atom-labelling scheme. Displacement ellipsoids are drawn at the 50% probability level.

## supplementary crystallographic information

### Comment

Research into transition metal complexes has been rapidly expanding because of their fascinating structural diversity, as well as their potential applications as functional materials and enzymes (Noro *et al.*, 2000; Yaghi *et al.*, 1998). And 4,4'-di-*tert*-butyl-2,2'-bipyridine has been used as a ligand in coordination chemistry (Huertas *et al.*, 2001; Qin *et al.*, 2002). We report here the crystal structure of the title copper(II)complex, (I), containing a bipyridine ligand.

In the crystal of (I), the Cu^II^ ion is coordinated by two N atoms of the 4,4'-di-*tert*-butyl-2,2'-bipyridine ligand and four O atoms from the two nitrate anions. The dihedral angle between the planes of two pyridine rings is 11.52 (10)°. The title compound forms intermolecular H bond whereas the protonated C1 atom act as hydrogen-bond donor and O4 atom act as hydrogen-bond acceptor, the distance of the C1—H1···O4 hydrogen bonds is 3.124 (3) Å (Table 1). Weak C—H···O interactions may help to establish the packing.

### Experimental

A solution of 4,4'-di-*tert*-butyl-2,2'-bipyridine (0.15 g, 0.56 mmol) in ethanol (50 ml) was added to a solution of Cu(N0_3_)_2_, (0.09 g, 0.56 mmol) in H_2_O (20 ml), and the resulting blue solution was stirred for 10 min at 313 K. Then, it was left to evaporate slowly at room temperature. After one week, blue crystals of (I) were isolated.

### Refinement

H atoms were placed in calculated positions and refined as riding, with C—H = 0.93- and 0.96 Å, and *U*_iso_(H) = 1.2–1.5*U*_eq_(C).

### Crystal data

**Table:**

[Cu(NO_3_)_2_(C_18_H_24_N_2_)]	*F*(000) = 948
*M**_r_* = 455.96	*D*_x_ = 1.442 Mg m^−^^3^
Orthorhombic, *P*2_1_2_1_2_1_	Mo *K*α radiation, λ = 0.71073 Å
Hall symbol: P 2ac 2ab	Cell parameters from 2120 reflections
*a* = 9.8265 (16) Å	θ = 2.0–25.0°
*b* = 13.247 (2) Å	µ = 1.08 mm^−^^1^
*c* = 16.138 (3) Å	*T* = 173 K
*V* = 2100.7 (6) Å^3^	Block, blue
*Z* = 4	0.27 × 0.25 × 0.17 mm

### Data collection

**Table:**

Bruker SMART CCD area-detector diffractometer	3642 independent reflections
Radiation source: fine-focus sealed tube	3450 reflections with *I* > 2σ(*I*)
graphite	*R*_int_ = 0.022
φ and ω scans	θ_max_ = 25.0°, θ_min_ = 2.0°
Absorption correction: multi-scan (*SADABS*; Bruker, 2005)	*h* = −11→11
*T*_min_ = 0.759, *T*_max_ = 0.837	*k* = −15→15
11065 measured reflections	*l* = −19→17

### Refinement

**Table:**

Refinement on *F*^2^	Secondary atom site location: difference Fourier map
Least-squares matrix: full	Hydrogen site location: inferred from neighbouring sites
*R*[*F*^2^ > 2σ(*F*^2^)] = 0.030	H-atom parameters constrained
*wR*(*F*^2^) = 0.074	*w* = 1/[σ^2^(*F*_o_^2^) + (0.0386*P*)^2^ + 0.8717*P*] where *P* = (*F*_o_^2^ + 2*F*_c_^2^)/3
*S* = 1.05	(Δ/σ)_max_ = 0.001
3642 reflections	Δρ_max_ = 0.64 e Å^−^^3^
258 parameters	Δρ_min_ = −0.81 e Å^−^^3^
1 restraint	Absolute structure: Flack (1983), 1522 Freidel pairs
Primary atom site location: structure-invariant direct methods	Flack parameter: 0.012 (13)

### Special details

**Table:**

Geometry. All e.s.d.'s (except the e.s.d. in the dihedral angle between two l.s. planes) are estimated using the full covariance matrix. The cell e.s.d.'s are taken into account individually in the estimation of e.s.d.'s in distances, angles and torsion angles; correlations between e.s.d.'s in cell parameters are only used when they are defined by crystal symmetry. An approximate (isotropic) treatment of cell e.s.d.'s is used for estimating e.s.d.'s involving l.s. planes.
Refinement. Refinement of *F*^2^ against ALL reflections. The weighted *R*-factor *wR* and goodness of fit *S* are based on *F*^2^, conventional *R*-factors *R* are based on *F*, with *F* set to zero for negative *F*^2^. The threshold expression of *F*^2^ > σ(*F*^2^) is used only for calculating *R*-factors(gt) *etc*. and is not relevant to the choice of reflections for refinement. *R*-factors based on *F*^2^ are statistically about twice as large as those based on *F*, and *R*- factors based on ALL data will be even larger.

### Fractional atomic coordinates and isotropic or equivalent isotropic displacement parameters (Å^2^)

**Table:**

	*x*	*y*	*z*	*U*_iso_*/*U*_eq_	
Cu1	0.58577 (3)	0.98172 (2)	0.72968 (2)	0.02515 (10)	
O2	0.3672 (2)	1.0193 (2)	0.66313 (14)	0.0434 (6)	
O1	0.4195 (2)	0.89792 (15)	0.74670 (14)	0.0387 (5)	
O4	0.5221 (2)	1.08313 (16)	0.81065 (13)	0.0328 (5)	
N2	0.7365 (2)	1.06673 (17)	0.68765 (15)	0.0243 (5)	
N4	0.5626 (2)	1.05068 (18)	0.88143 (15)	0.0300 (6)	
O5	0.6358 (2)	0.97414 (17)	0.88213 (13)	0.035	
N1	0.6896 (2)	0.87411 (17)	0.67467 (15)	0.0242 (5)	
N3	0.3281 (3)	0.9442 (2)	0.70369 (17)	0.0381 (6)	
O6	0.5273 (3)	1.0950 (2)	0.94430 (15)	0.0558 (7)	
C15	1.0722 (3)	1.2220 (2)	0.56813 (19)	0.0308 (7)	
C6	0.8337 (3)	1.0146 (2)	0.64671 (16)	0.0225 (6)	
C11	0.9809 (3)	0.6535 (2)	0.58589 (19)	0.0279 (6)	
O3	0.2114 (2)	0.9132 (2)	0.7046 (2)	0.0649 (9)	
C2	0.7514 (3)	0.7040 (2)	0.64459 (18)	0.0275 (6)	
H2	0.7265	0.6363	0.6436	0.033*	
C3	0.8801 (3)	0.7323 (2)	0.61767 (18)	0.0233 (6)	
C8	0.9527 (3)	1.1675 (2)	0.61021 (18)	0.0253 (6)	
C7	0.9411 (3)	1.0624 (2)	0.60741 (17)	0.0252 (6)	
H7	1.0057	1.0245	0.5791	0.030*	
C5	0.8133 (3)	0.9040 (2)	0.64572 (17)	0.0225 (6)	
C4	0.9100 (3)	0.83539 (19)	0.61865 (16)	0.0223 (5)	
H4	0.9948	0.8580	0.6011	0.027*	
C1	0.6602 (3)	0.7753 (2)	0.67289 (19)	0.0281 (6)	
H1	0.5752	0.7541	0.6914	0.034*	
C10	0.7481 (3)	1.1682 (2)	0.69174 (19)	0.0290 (6)	
H10	0.6821	1.2047	0.7202	0.035*	
C17	1.1679 (4)	1.2577 (3)	0.6369 (2)	0.0507 (10)	
H17A	1.2445	1.2919	0.6128	0.076*	
H17B	1.1993	1.2006	0.6681	0.076*	
H17C	1.1202	1.3032	0.6730	0.076*	
C9	0.8536 (3)	1.2190 (2)	0.65550 (19)	0.0302 (7)	
H9	0.8594	1.2888	0.6611	0.036*	
C14	1.1222 (3)	0.6979 (3)	0.5698 (2)	0.0400 (8)	
H14A	1.1817	0.6459	0.5498	0.060*	
H14B	1.1580	0.7251	0.6205	0.060*	
H14C	1.1155	0.7507	0.5292	0.060*	
C13	0.9948 (3)	0.5689 (2)	0.6509 (2)	0.0397 (8)	
H13A	1.0577	0.5189	0.6312	0.060*	
H13B	0.9076	0.5381	0.6600	0.060*	
H13C	1.0276	0.5969	0.7020	0.060*	
C16	1.1495 (3)	1.1522 (3)	0.5092 (2)	0.0410 (8)	
H16A	1.2235	1.1885	0.4844	0.062*	
H16B	1.0890	1.1287	0.4667	0.062*	
H16C	1.1845	1.0955	0.5396	0.062*	
C12	0.9251 (4)	0.6105 (3)	0.5041 (2)	0.0440 (8)	
H12A	0.9871	0.5607	0.4829	0.066*	
H12B	0.9154	0.6641	0.4645	0.066*	
H12C	0.8380	0.5798	0.5139	0.066*	
C18	1.0207 (4)	1.3135 (3)	0.5182 (3)	0.0512 (10)	
H18A	1.0965	1.3467	0.4923	0.077*	
H18B	0.9755	1.3598	0.5547	0.077*	
H18C	0.9581	1.2910	0.4764	0.077*	

### Atomic displacement parameters (Å^2^)

**Table:**

	*U*^11^	*U*^22^	*U*^33^	*U*^12^	*U*^13^	*U*^23^
Cu1	0.02290 (16)	0.02379 (17)	0.02875 (18)	0.00229 (14)	0.00540 (15)	−0.00019 (15)
O2	0.021	0.0581 (15)	0.0509 (13)	0.0047 (11)	−0.0042 (9)	0.0038 (14)
O1	0.0287 (10)	0.0327 (11)	0.0548 (15)	−0.0013 (9)	0.0091 (10)	0.0042 (9)
O4	0.0379 (11)	0.0321 (12)	0.0285 (11)	0.0100 (9)	0.0039 (10)	0.0011 (10)
N2	0.0264 (12)	0.0209 (12)	0.0256 (12)	0.0016 (10)	0.0028 (10)	0.0006 (10)
N4	0.0287 (13)	0.0328 (14)	0.0283 (13)	0.0002 (11)	0.0005 (11)	−0.0032 (11)
O5	0.033	0.034	0.038	0.0127 (10)	−0.0048 (9)	0.0036 (10)
N1	0.0248 (11)	0.0220 (13)	0.0260 (13)	−0.0020 (10)	0.0038 (10)	0.0000 (10)
N3	0.0263 (13)	0.0424 (16)	0.0455 (17)	0.0029 (11)	0.0057 (10)	−0.0132 (13)
O6	0.0737 (18)	0.0623 (17)	0.0313 (14)	0.0154 (14)	0.0022 (13)	−0.0100 (12)
C15	0.0309 (16)	0.0254 (15)	0.0361 (17)	−0.0065 (14)	−0.0001 (15)	0.0046 (12)
C6	0.0253 (13)	0.0220 (14)	0.0201 (13)	0.0005 (12)	0.0013 (11)	0.0000 (12)
C11	0.0330 (15)	0.0207 (15)	0.0299 (16)	0.0024 (13)	0.0008 (13)	−0.0045 (12)
O3	0.0275 (13)	0.0736 (19)	0.094 (2)	−0.0068 (12)	−0.0036 (13)	−0.0171 (17)
C2	0.0291 (14)	0.0201 (14)	0.0332 (17)	−0.0039 (12)	0.0012 (13)	−0.0010 (12)
C3	0.0286 (16)	0.0214 (14)	0.0200 (14)	0.0008 (11)	−0.0055 (12)	0.0007 (11)
C8	0.0287 (15)	0.0230 (15)	0.0242 (16)	−0.0037 (11)	−0.0047 (12)	0.0036 (12)
C7	0.0239 (15)	0.0255 (14)	0.0262 (15)	−0.0011 (11)	0.0027 (12)	−0.0024 (12)
C5	0.0246 (13)	0.0227 (15)	0.0202 (14)	−0.0013 (11)	0.0003 (12)	−0.0016 (12)
C4	0.0206 (12)	0.0243 (14)	0.0218 (13)	−0.0023 (12)	0.0018 (13)	−0.0016 (11)
C1	0.0258 (15)	0.0240 (15)	0.0344 (17)	−0.0039 (12)	0.0010 (13)	−0.0002 (13)
C10	0.0345 (15)	0.0232 (15)	0.0295 (16)	0.0059 (13)	0.0041 (13)	−0.0012 (12)
C17	0.045 (2)	0.056 (2)	0.051 (2)	−0.0223 (18)	−0.0037 (18)	−0.0033 (19)
C9	0.0381 (16)	0.0187 (15)	0.0339 (17)	−0.0004 (12)	−0.0017 (14)	−0.0016 (13)
C14	0.0333 (18)	0.0337 (18)	0.053 (2)	0.0072 (14)	0.0107 (15)	−0.0022 (16)
C13	0.0459 (19)	0.0285 (17)	0.045 (2)	0.0100 (15)	−0.0019 (16)	0.0064 (15)
C16	0.0365 (17)	0.0393 (19)	0.047 (2)	−0.0091 (15)	0.0115 (16)	0.0047 (16)
C12	0.051 (2)	0.043 (2)	0.0384 (18)	0.0096 (18)	−0.0011 (17)	−0.0151 (14)
C18	0.047 (2)	0.040 (2)	0.067 (3)	−0.0033 (17)	0.0112 (19)	0.0231 (19)

### Geometric parameters (Å, °)

**Table:**

Cu1—N1	1.965 (2)	C8—C9	1.396 (4)
Cu1—O4	1.976 (2)	C8—C7	1.398 (4)
Cu1—N2	1.980 (2)	C7—H7	0.9300
Cu1—O1	1.994 (2)	C5—C4	1.385 (4)
O2—N3	1.251 (4)	C4—H4	0.9300
O1—N3	1.290 (3)	C1—H1	0.9300
O4—N4	1.284 (3)	C10—C9	1.368 (4)
N2—C6	1.351 (3)	C10—H10	0.9300
N2—C10	1.350 (4)	C17—H17A	0.9600
N4—O6	1.223 (3)	C17—H17B	0.9600
N4—O5	1.243 (3)	C17—H17C	0.9600
N1—C1	1.340 (4)	C9—H9	0.9300
N1—C5	1.360 (4)	C14—H14A	0.9600
N3—O3	1.218 (3)	C14—H14B	0.9600
C15—C16	1.528 (4)	C14—H14C	0.9600
C15—C17	1.531 (5)	C13—H13A	0.9600
C15—C8	1.536 (4)	C13—H13B	0.9600
C15—C18	1.541 (4)	C13—H13C	0.9600
C6—C7	1.385 (4)	C16—H16A	0.9600
C6—C5	1.479 (4)	C16—H16B	0.9600
C11—C3	1.528 (4)	C16—H16C	0.9600
C11—C14	1.530 (4)	C12—H12A	0.9600
C11—C12	1.539 (4)	C12—H12B	0.9600
C11—C13	1.541 (4)	C12—H12C	0.9600
C2—C1	1.380 (4)	C18—H18A	0.9600
C2—C3	1.389 (4)	C18—H18B	0.9600
C2—H2	0.9300	C18—H18C	0.9600
C3—C4	1.396 (4)		
			
N1—Cu1—O4	163.03 (9)	C4—C5—C6	124.1 (2)
N1—Cu1—N2	82.49 (9)	C5—C4—C3	120.0 (3)
O4—Cu1—N2	94.41 (9)	C5—C4—H4	120.0
N1—Cu1—O1	94.82 (9)	C3—C4—H4	120.0
O4—Cu1—O1	91.59 (9)	N1—C1—C2	122.4 (3)
N2—Cu1—O1	167.53 (10)	N1—C1—H1	118.8
N3—O1—Cu1	103.37 (17)	C2—C1—H1	118.8
N4—O4—Cu1	105.23 (16)	N2—C10—C9	122.2 (3)
C6—N2—C10	118.2 (2)	N2—C10—H10	118.9
C6—N2—Cu1	113.90 (18)	C9—C10—H10	118.9
C10—N2—Cu1	127.8 (2)	C15—C17—H17A	109.5
O6—N4—O5	123.3 (3)	C15—C17—H17B	109.5
O6—N4—O4	119.3 (2)	H17A—C17—H17B	109.5
O5—N4—O4	117.4 (2)	C15—C17—H17C	109.5
C1—N1—C5	118.0 (2)	H17A—C17—H17C	109.5
C1—N1—Cu1	127.3 (2)	H17B—C17—H17C	109.5
C5—N1—Cu1	114.09 (18)	C10—C9—C8	120.8 (3)
O3—N3—O2	124.3 (3)	C10—C9—H9	119.6
O3—N3—O1	119.2 (3)	C8—C9—H9	119.6
O2—N3—O1	116.5 (2)	C11—C14—H14A	109.5
C16—C15—C17	109.4 (3)	C11—C14—H14B	109.5
C16—C15—C8	111.8 (2)	H14A—C14—H14B	109.5
C17—C15—C8	107.1 (3)	C11—C14—H14C	109.5
C16—C15—C18	108.3 (3)	H14A—C14—H14C	109.5
C17—C15—C18	109.7 (3)	H14B—C14—H14C	109.5
C8—C15—C18	110.5 (3)	C11—C13—H13A	109.5
N2—C6—C7	121.9 (3)	C11—C13—H13B	109.5
N2—C6—C5	114.6 (2)	H13A—C13—H13B	109.5
C7—C6—C5	123.5 (3)	C11—C13—H13C	109.5
C3—C11—C14	112.4 (2)	H13A—C13—H13C	109.5
C3—C11—C12	108.1 (3)	H13B—C13—H13C	109.5
C14—C11—C12	108.7 (3)	C15—C16—H16A	109.5
C3—C11—C13	109.0 (2)	C15—C16—H16B	109.5
C14—C11—C13	108.4 (3)	H16A—C16—H16B	109.5
C12—C11—C13	110.3 (3)	C15—C16—H16C	109.5
C1—C2—C3	120.6 (3)	H16A—C16—H16C	109.5
C1—C2—H2	119.7	H16B—C16—H16C	109.5
C3—C2—H2	119.7	C11—C12—H12A	109.5
C2—C3—C4	116.9 (3)	C11—C12—H12B	109.5
C2—C3—C11	120.7 (2)	H12A—C12—H12B	109.5
C4—C3—C11	122.4 (3)	C11—C12—H12C	109.5
C9—C8—C7	116.5 (3)	H12A—C12—H12C	109.5
C9—C8—C15	122.4 (3)	H12B—C12—H12C	109.5
C7—C8—C15	121.0 (3)	C15—C18—H18A	109.5
C6—C7—C8	120.2 (3)	C15—C18—H18B	109.5
C6—C7—H7	119.9	H18A—C18—H18B	109.5
C8—C7—H7	119.9	C15—C18—H18C	109.5
N1—C5—C4	122.0 (3)	H18A—C18—H18C	109.5
N1—C5—C6	113.9 (2)	H18B—C18—H18C	109.5

### Hydrogen-bond geometry (Å, °)

**Table:**

*D*—H···*A*	*D*—H	H···*A*	*D*···*A*	*D*—H···*A*
C1—H1···O4^i^	0.93	2.46	3.124 (3)	129

Symmetry codes: (i) −*x*+1, *y*−1/2, −*z*+3/2.
